# Aesthetic and Functional Rehabilitation for Dentogingival Asymmetry Using Zirconia Restorations

**DOI:** 10.7759/cureus.63558

**Published:** 2024-07-01

**Authors:** Nisarga R Mahajan, Akansha Bansod, Amit Reche, Surekha A Dubey

**Affiliations:** 1 Department of Prosthodontics, Sharad Pawar Dental College and Hospital, Datta Meghe Institute of Higher Education and Research, Wardha, IND; 2 Department of Public Health Dentistry, Sharad Pawar Dental College and Hospital, Datta Meghe Institute of Higher Education and Research, Wardha, IND

**Keywords:** aesthetic rehabilitation, management, treatment, anterior restoration, zirconia crown

## Abstract

In an effort to improve dentogingival aesthetics, scientific research has been developing non-surgical techniques and dental restorative materials. A 45-year-old female reported to the hospital with a complaint of an unpleasant aesthetic in the anterior region of the maxilla. She has an irregular contour of the gingiva in her anterior region and no temporomandibular problems. There is a history of trauma, but she did not take any treatment for it, which caused discolouration. Plaque index was retained by the surface roughness. Due to this reason, the patient's appearance, self-esteem, and quality of life have decreased. The purpose of this case is to provide a system-based clinical example of oral rehabilitation for anterior teeth. The patient didn't like the way she looked, and hence aesthetic and functional rehabilitation was planned. Treating the dentogingival asymmetry was the first step in the therapeutic approach. An in-ceram zirconia permanent porcelain metal-free crown was positioned after the temporary crowns were cemented into place. It should be mentioned that in clinical situations, the integration of periodontal and prosthetic treatment is crucial. Additionally, it is stated that the in-ceram zirconia system can produce satisfactory results when applied appropriately. For the main anterior teeth, zirconia crowns have offered an alternate treatment option that addresses aesthetic concerns and facilitates the installation of extra-coronal restorations. This article describes a case involving the aesthetic and functional restoration of severely damaged maxillary incisors using zirconia crowns.

## Introduction

A pleasant smile is the initial point of contact between individuals; repairing an unattractive smile in the maxilla is always a therapeutic challenge, especially when the teeth are not shaped and proportioned properly [1]. In addition, if the shade is unsightly, the restoration is outdated [2]. Clinical judgement is one of the most crucial parts of clinical dentistry for tooth restoration [3], photographs, and diagnostic wax-up examinations. Dental professionals and patients now have additional options because of technological developments and a shift towards more conservative treatments. A useful tool that makes it easier to complete a comprehensive assessment of the patient's dental and facial features is digital smile design (DSD) [4]. The discovery of morphological differences between soft and hard tissues is made possible by this research. Moreover, as the patient's expectations are primarily based on aesthetic results [5], careful coordination between the dental specialist and dental lab staff is necessary to achieve those goals. Because of their longevity and effectiveness, all-ceramic systems are used in oral rehabilitation all over the world, taking into account their capacity to replicate the colour, translucency, and roughness of a tooth's surface.

Over the past forty years, porcelain fused to metal crowns, which had aesthetic drawbacks, have been replaced by all-ceramic crowns. Different types of materials have restrictions both in terms of appearance and functionality, and all ceramic crowns can be created to fit them [6]. This article describes a clinical example where metal-free ceramic crowns are cemented, the gingiva was treated with provisional restorations using a dynamic compression approach, and the anterior maxillary prosthesis was planned using Exocad software (Exocad Dental CAD software, Darmstadt, Germany).

## Case presentation

**Figure 1:**
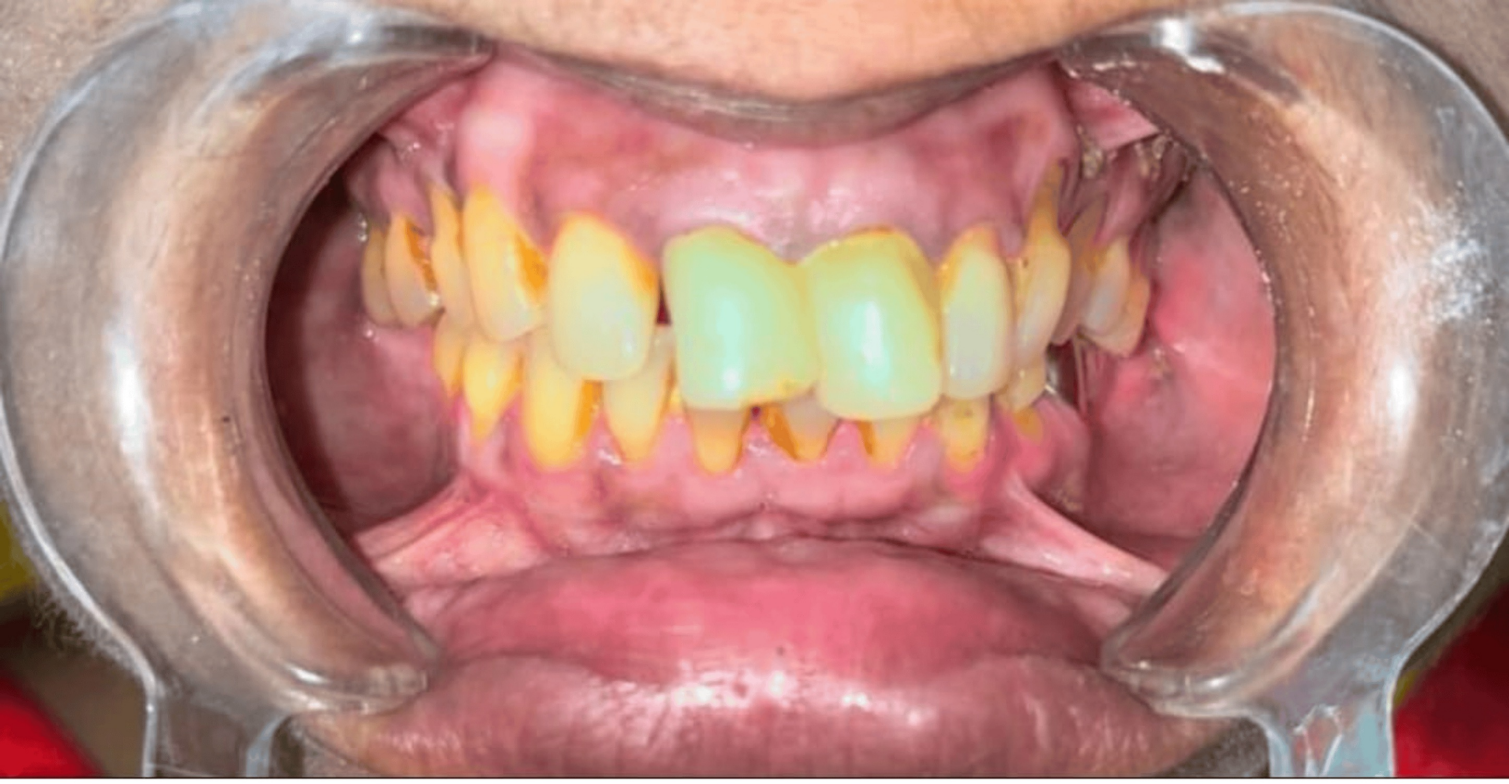
Preoperative photo showing discoloured and poor aesthetic.

The case report shows a 45-year-old female patient who visited the hospital for poor aesthetics in the anterior region, in which she was unhappy, as shown in Figure 1. The gingival contour and tooth axis were found to be unsuitable at first.

**Figure 2:**
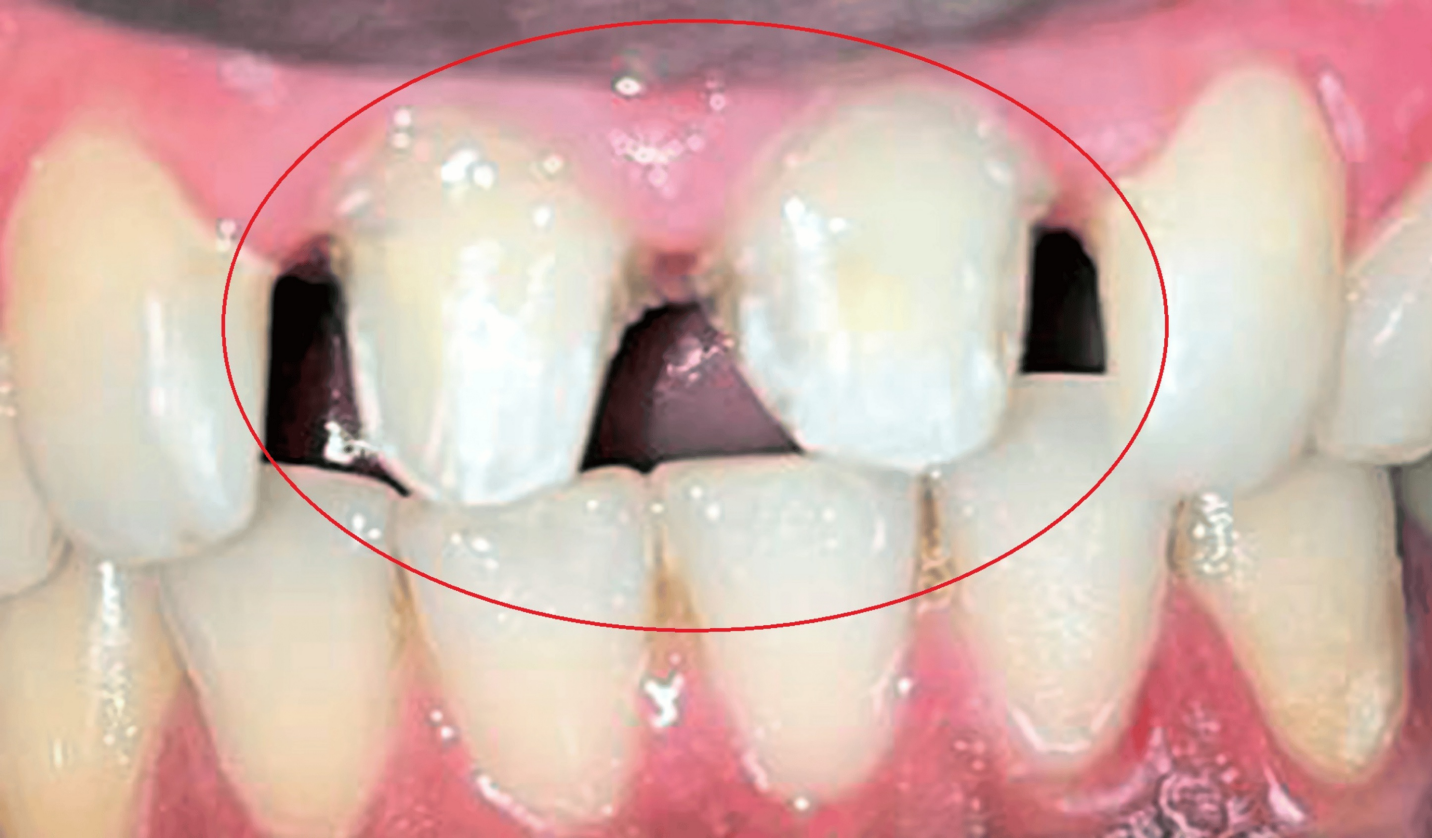
Tooth preparation with tooth number 11, 12.

She did not reveal any symptoms of temporomandibular joint dysfunction on palpation and used the bimanual technique. Her previous trauma has been well documented, but she never received treatment for it, which resulted in discolouration of the anterior teeth, and on examination, the teeth indicated a negative vitality pulp test. The patient had a strong desire to make her smile seem better. Planned root canal treatment was performed in the anterior region (tooth number 11, 12) to correct proclination in addition to tooth preparation, as shown in Figure 2.

**Figure 3:**
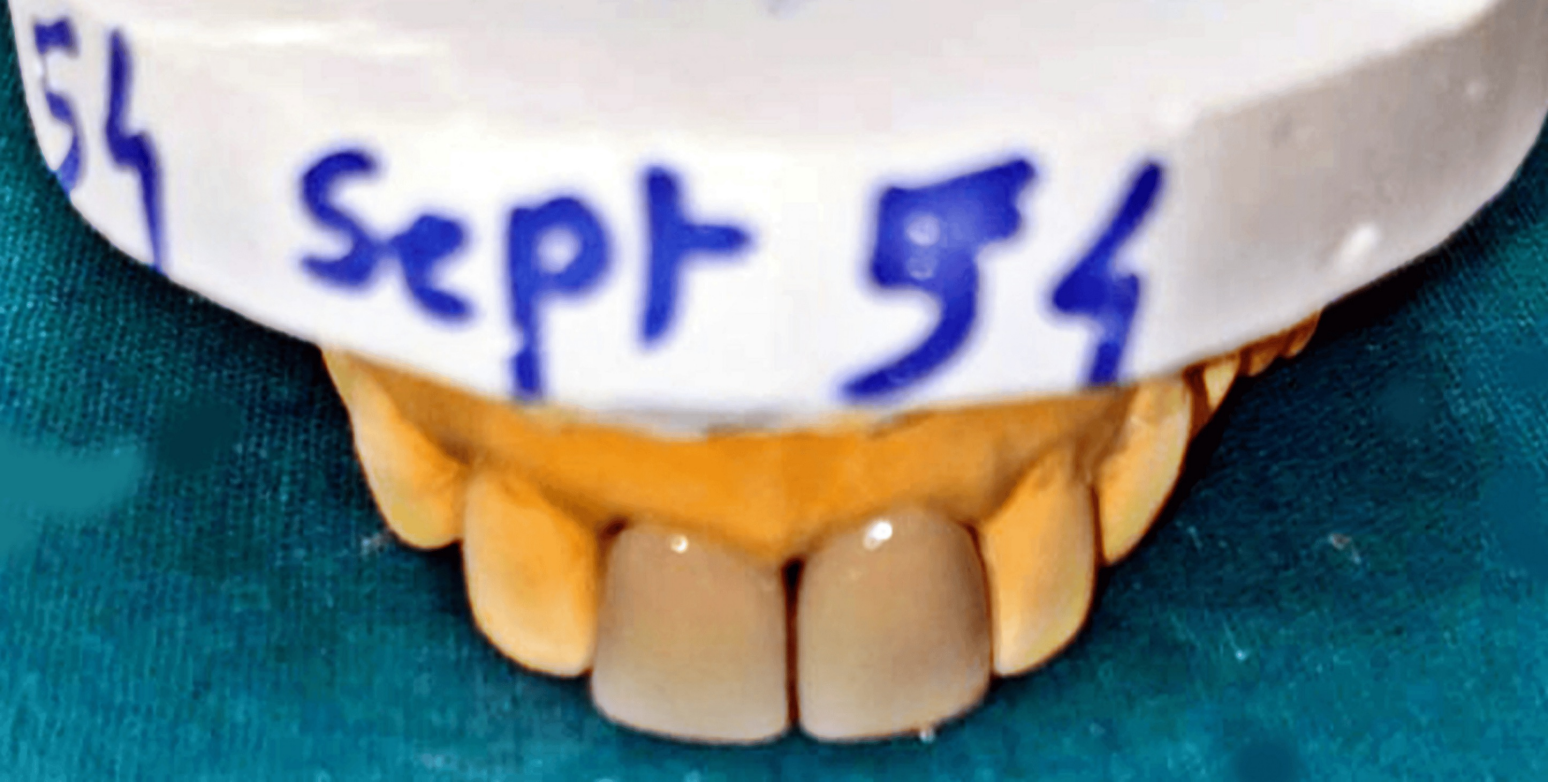
Temporary prosthesis on cast.

The finish line prepared was a deep chamfer with softer interior line angles. The preparation involved the use of diamond burs with the bi-planar reduction on the labial surface to correct the inclination profile for all-ceramic restorations; the incisal reduction was made up to 1.5 mm, and the labial and lingual reduction up to 1.0-1.5 mm. Next, an irreversible hydrocolloid (Zhermack Tropical Alginate Company, Germany) was used to make a primary maxilla impression. Dental stone type 3 (Neelkanth Dental Stone Plaster Class III, Jodhpur, Rajasthan) was used to create the study cast. Additionally, a photographic methodology was developed that made it possible to analyse the smile and plan the colour, size, arrangement, horizontal reference plane, and facial midline in great detail. Based on aesthetic considerations, the anterior area of the maxilla was modified, and the marginal gingival contour was modified as per the aesthetic requirements. The tooth contour was modified in the prosthetic design. Furthermore, there were irregularities in the height of the incisor as well as the interproximal papilla, and the interproximal contacts were poor, as shown in Figure 3.

**Figure 4:**
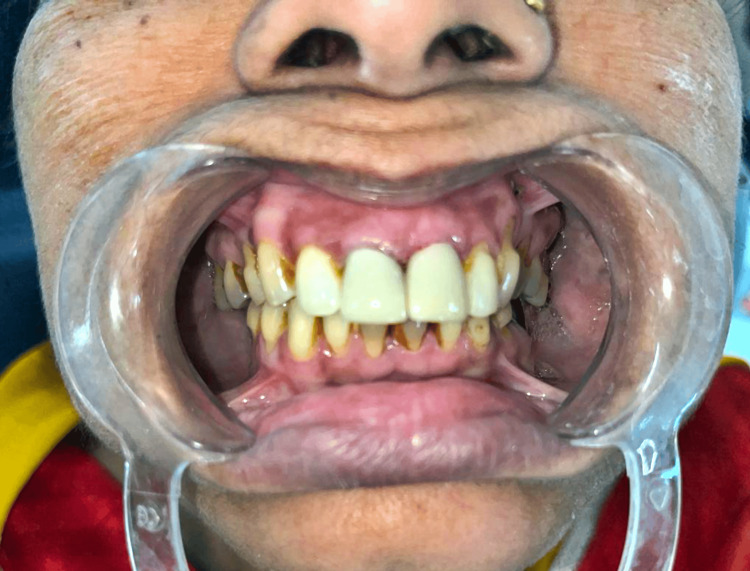
Temporary prosthesis in patient's oral cavity.

The results of a diagnostic wax-up used to visualise the planned treatment are shown in Figure 3. In addition to matching shade, the optimal dimensions, form, and contour of the maxillary anterior region were noted. The patient verified the diagnostic wax-up to ensure that the treatment plan was correct. To satisfy occlusal aesthetic standards, the form, size, and colour of these temporary prostheses were carefully refined as shown in Figure 4.

**Figure 5:**
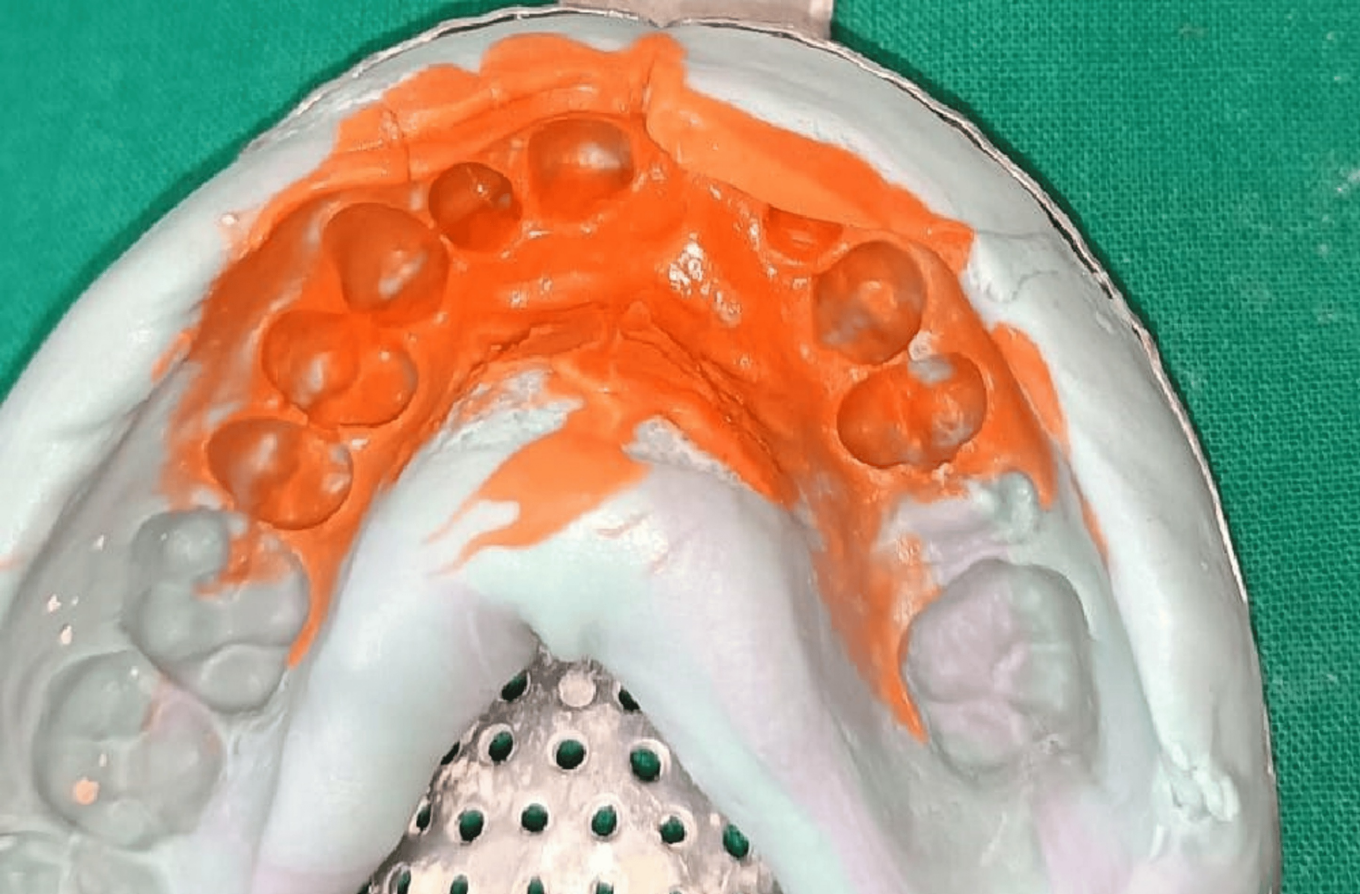
Final impression with polyvinyl siloxane impression material.

Functional mandibular movements were evaluated, and anterior guidance was given so that the functions were not hampered. This temporary prosthesis worked really well for the patient. The next step was to make the final impression. The addition of silicone, or polyvinyl siloxane (Coltene Affinis Set, New Delhi), was used to make the final impression as shown in Figure 5.

**Figure 6:**
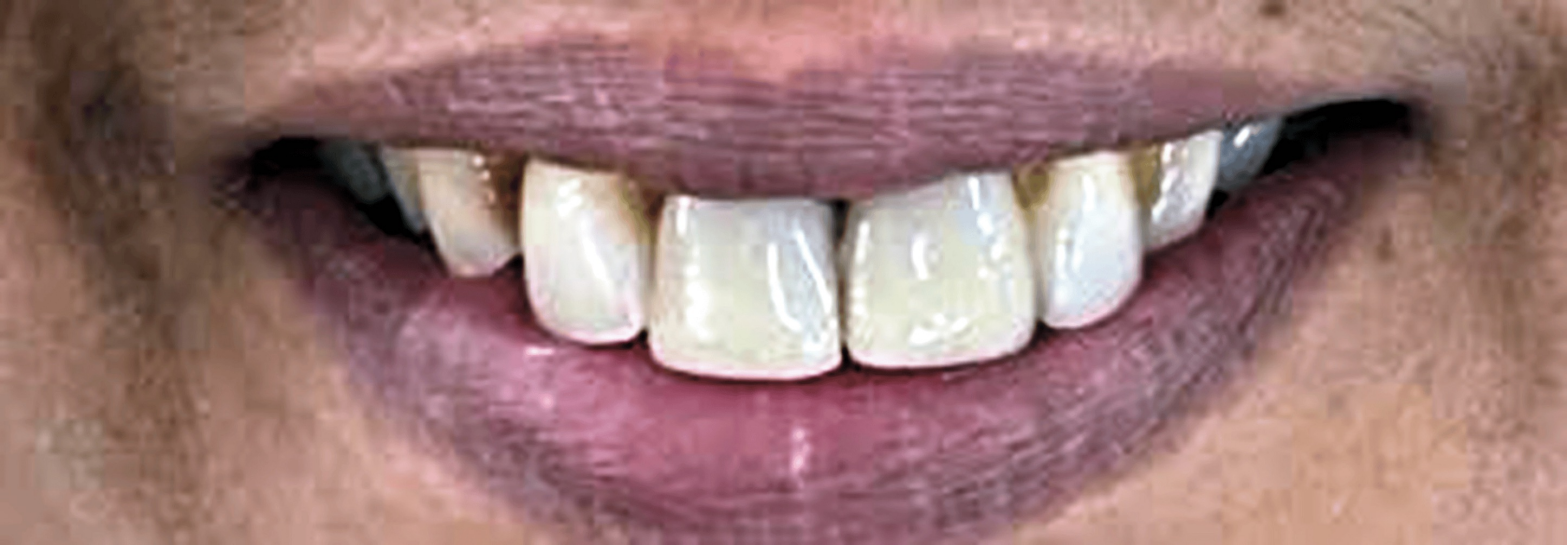
Post-operative photo with aesthetic and functional rehabilitation.

Impression was made using the addition of polyvinyl siloxane, as it exhibits little distortion and high dimensional stability. It can also result in a final cast that is correct and hence, this was the material of choice for making final impressions. The computer-aided design/computer-aided manufacturing (CAD/CAM) method was used to create prostheses; an impression was taken and subsequently transmitted to the lab. After scanning their functional cast using a lab scanner (Sirona Dentsply, Charlotte, North Carolina), the design of the restoration was done on Exocad software, and computer-aided manufacturing was used to construct the crown. The course of treatment, which included zirconia crown (Dental Direkt, Spenge, Germany) restorations for all maxillary anterior teeth, has left the patient satisfied. The insertion, margin, fit, and retention of the zirconia crowns were evaluated and crowns were cemented by using resin adhesive cement (Dentsply Calibra Universal Self-adhesive Resin Cement, Charlotte, North Carolina). After that, any extra cement was carefully scraped off. The patient's function and appearance have been restored and she is satisfied with the treatment, as seen as shown in Figure 6.

Periodic recalls were planned after a month and then every six months.

## Discussion

Deterioration of the teeth usually requires prolonged dental work. It is necessary to consider which therapy will help the patient for the longest period of time, have the most acceptable lifespan, and offer the highest overall value in terms of expenses. The choice of material should be based on both strength and aesthetics, which directly have a major influence on the length of restorations and the predictability of therapy [7]. Because patients and dentists have increased expectations for cosmetic restorations, metal-free restorations are becoming more and more popular as an alternative to metal-ceramic restorations [8,9]. Because of their superior aesthetics, biocompatibility, and long-lasting restorations, ceramic systems are a great substitute for crown restorations and are being used more and more successfully for anterior tooth rehabilitation [10]. Wax-ups are frequently utilised in treatment planning to help create a visually pleasing and well-functioning environment. But in this instance, digital smile design (DSD) was first used to improve communication between the patient and the technician and provide an aesthetically pleasing diagnosis [11]. Many ceramic procedures have been developed for clinical usage, and ceramic restorations are frequently employed in the rehabilitation of anterior and posterior teeth [12]. Zirconia-based dental ceramics have superior mechanical strength properties compared to traditional glass-ceramic restorations [13].

When anterior teeth were restored using zirconia-based tooth-supported crowns, favourable clinical outcomes were attained [14,15]. CAD/CAM technology is now used in dental offices to create zirconia, which has significant mechanical and physical qualities like high strength, sufficient toughness, biocompatibility, and visually acceptable results [16,17]. Using monolithic zirconia restorations should result in improved clinical performance [18,19]. It has also been demonstrated to lead to excellent aesthetic standards and a decrease in the quantity of metal used in the mouth [20]. According to a recent study, zirconia exhibited some translucency and was less susceptible to thickness than porcelain free of leucite and lithium disilicate. But when the thickness shrank, the zirconia ceramics' translucency also rose rapidly [21]. Anterior zirconium veneer cementation is a critical phase in the entire technological process, and it is essential to choose a material that does not include tertiary amine, as does the majority of light-curing cement. Conversely, while working with zirconium, a dual-polymerized fixating agent that is amine-reduced, amine-free, and devoid of benzoyl peroxide yields superior binding strength and improves colour stability [22]. To forecast the outcome of an oral treatment, especially where aesthetics is involved, a clear diagnosis and a thorough treatment plan that takes into account each specific element of the face and dentogingival system should be required [23].

## Conclusions

Given the patient's great respect for aesthetics, an all-ceramic restoration-especially a multilayer zirconia restoration-was the suggested course of therapy. CAD/CAM zirconia prosthetic teeth offer uniform colour and long-lasting beauty, along with improved biocompatibility and a decreased likelihood of wearing down nearby teeth. Zirconia restorations have superior mechanical, chemical, and clinical properties, making them a viable substitute material in the field of prosthodontics. In summary, zirconia restorations in the maxillary front area provide a useful means of achieving goals related to appearance and functionality. Through this case study, we have demonstrated the advantages of zirconia, such as its hardness, biocompatibility, and aesthetic similarity to real teeth. The patient's grin has been effectively restored with zirconia crowns, indicating the material's promise as a practical remedy for cosmetic problems with the front area. An effective substitute for anterior dental restorations are nanocomposites. Additionally, excellent planning produces very pleasing outcomes that make it easier to repair an improper axial tilt of the incisors. When combined with a precise treatment plan, zirconia-containing ceramic restorations enable the practitioner to successfully combine form and function for good clinical outcomes.
